# An infant with drug reaction with eosinophilia and systemic symptoms caused by phenobarbital and amoxicillin: A case report

**DOI:** 10.1097/MD.0000000000045034

**Published:** 2025-10-10

**Authors:** Yurika Matsumoto, Mayumi Fujita, Chisato Inuo

**Affiliations:** a Department of Allergy, Kanagawa Children’s Medical Center, Kanagawa, Japan.

**Keywords:** amoxicillin, drug provocation test, drug reaction with eosinophilia and systemic symptoms, infant, phenobarbital

## Abstract

**Rationale:**

Drug reaction with eosinophilia and systemic symptoms (DRESS) is rare in children, and reports of cases associated with amoxicillin (AMX), the most commonly prescribed antibiotic in children, are extremely rare. Diagnosing DRESS in infants is particularly challenging owing to its rarity and atypical symptoms, often leading to delays in diagnosis and appropriate management. Furthermore, there is insufficient reporting on the role of AMX as a trigger for DRESS in children, raising concerns about diagnostic delays.

**Patient concerns:**

A 12-month-old girl experienced 3 discrete episodes over 11 months after exposure to phenobarbital and later AMX, with fever, rash, eosinophilia, facial edema, and purpura.

**Diagnoses:**

Recurrent DRESS attributed to phenobarbital (episode 1) and AMX (episodes 2–3), supported by positive patch tests to both drugs, a positive AMX drug provocation test, and a markedly elevated thymus and activation-regulated chemokine level in episode 3 (33,925 pg/mL); RegiSCAR scores were 5, 4, and 3, respectively.

**Interventions:**

In episodes 1 and 3, the patient improved with supportive therapy only. In episode 2, the patient was treated with steroids for wheezing before being suspected of DRESS symptoms.

**Outcomes:**

The patient recovered fully without sequelae.

**Lessons:**

This case underscores the need for vigilance in diagnosing DRESS in infants, even with common antibiotics like AMX, and highlights the need for further pediatric research.

## 1. Introduction

Drug reaction with eosinophilia and systemic symptoms (DRESS) is a severe drug-induced hypersensitivity syndrome. It is characterized by fever, rash, eosinophilia, and multi-organ dysfunction, such as lymphadenopathy and hepatitis. Although aromatic antiepileptics are common triggers in adults,^[1]^ in children, DRESS is uncommon, and documented cases involving amoxicillin (AMX) – the most frequently prescribed antibiotic for children – are exceptionally rare.^[2,3]^ Diagnosing DRESS in infants is particularly challenging owing to its rarity and atypical presentation, often leading to delayed recognition and management. Furthermore, the role of AMX as a trigger in pediatric patients with DRESS remains underreported, raising concerns about underdiagnosis.

Pediatric cases of recurrent AMX-triggered DRESS after re-exposure have rarely been documented. Herein, we report a case of recurrent DRESS in a 12-month-old infant in whom causality was confirmed by both a positive patch test and a positive drug provocation test (DPT).

## 2. Case report

### 2.1. The first episode

A 12-month-old girl with a history of preterm birth and low birth weight was treated with ampicillin hydrate for 5 days. Subsequently, phenobarbital (PB) was administered for 4 days to maintain sedation during intubation. Eighteen days after stopping ampicillin hydrate and 9 days after the last dose of PB, she developed a fever and erythema. Laboratory tests showed eosinophilia (875/μL [white cell count: 39.8 × 10³/μL, 2.2% eosinophils]), and elevated liver enzymes (aspartate aminotransferase 105 units/L, alanine aminotransferase 118 units/L. Age-appropriate reference ranges are provided in Table 1). The patient met the European Registry of Severe Cutaneous Adverse Reactions (RegiSCAR) criteria for probable DRESS (score of 5)^[5,6]^ and improved with supportive care only.

**Table 1:** Features of the 3 episodes.

	First episode	Second episode	Third episode	Reference range (for age)
Age	12 mo	18 mo	23 mo	
Drug administration period				
Phenobarbital	4 d	N/A	N/A	
Ampicillin hydrate	5 d	2 d	N/A	
Amoxicillin	N/A	Single dose	Single dose	
Period from the last administration of ampicillin hydrate or amoxicillin to the onset of symptoms	18 d	3 d	7 h	
Clinical features	Fever, erythema	Rash	Fever, generalized rash, facial edema, purpura	
Laboratory findings				
WBC (/μL)	39,800	19,800	8300	6000–17,500
Maximum eosinophil count (/μL)	875	3267	1120	0–500
AST/ALT (U/L)	105/118	28/15	31/14	22–50/5–31
CRP (mg/dL)	2.0	0.6	4.26	<0.30
TARC (pg/mL)	NP	NP	33,925	0–1431 (<2 yr), cut off 13,900 for DRESS
Human herpesvirus 6	NP	NP	PCR−/EIA IgG ≥ 256	
Diagnostic score				
RegiSCAR DRESS score (points)*	5 (probable)	4 (probable)	3 (possible)	
Confirmatory testing				
Patch test			Positive (PB, AMX)	
Outcome	Supportive only	Corticosteroids were administered for wheezing	Supportive only	

Reference ranges: WBC, eosinophil count, AST/ALT, CRP – institutional pediatric laboratory standards, TARC.^[4]^

ALT = alanine aminotransferase, AMX = amoxicillin, AST = aspartate aminotransferase, CRP = C-reactive protein, HHV-6 = human herpesvirus-6, IgG = immunoglobulin N/A = not applicable, NP = not performed, PB = phenobarbital, PCR = polymerase chain reaction, RegiSCAR = European Registry of Severe Cutaneous Adverse Reactions, TARC = thymus and activation-regulated chemokine, WBC = white blood cell.

* Please see Table S1, Supplemental Digital Content 1, https://links.lww.com/MD/Q288.

### 2.2. The second episode

At 18 months of age, after 2 days of ampicillin hydrate and a single dose of AMX, she developed a rash and marked eosinophilia (3267/μL) with a RegiSCAR score of 4. She was treated with steroids for wheezing before DRESS was suspected. Only supportive therapy was provided for DRESS symptoms.

### 2.3. The third episode

At 23 months of age, she underwent a DPT with AMX. Seven hours after the first single dose, she developed a fever and a generalized rash. Laboratory tests showed eosinophilia (1120/μL [white cell count: 8.3 × 10^3^/μL, 13% eosinophils]) and elevated C-reactive protein level (4.26 mg/dL). Facial edema and purpura were also noted. Her RegiSCAR score was 3 for this episode. Treatment consisted of supportive care only.

**Figure 1.**
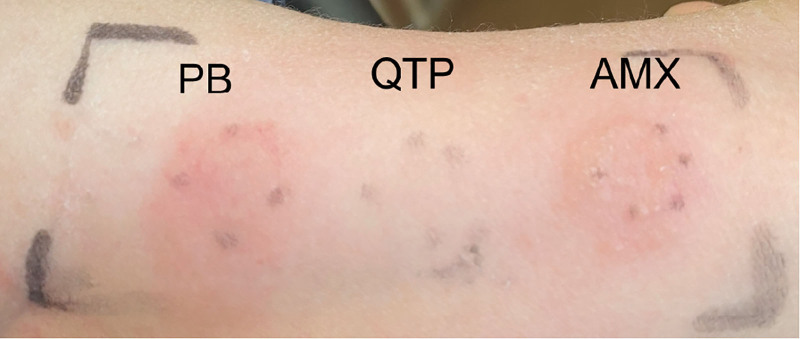
Patch tests were conducted for phenobarbital (PB), quetiapine (QTP), and amoxicillin (AMX). AMX = amoxicillin, PB = phenobarbital, QTP = quetiapine.

In episode 3, virology and biomarker testing were performed: HHV-6 real-time polymerase chain reaction (PCR) was negative with antibody titers of ≥256, and the serum thymus and activation-regulated chemokine (TARC) level was markedly elevated (33,925 pg/mL). These investigations were not performed during episodes 1 and 2. Skin testing showed positive patch test reactions to PB and AMX (Fig. 1). In vitro testing included lymphocyte transformation test (LTT) for AMX 3 days after the AMX DPT and LTT for PB during the recovery period; both were negative. A patch test for ampicillin hydrate was not undertaken because ampicillin was not initially suspected as the causative agent. Table 1 compares the clinical features, laboratory values, and outcomes across all 3 episodes, and the RegiSCAR scoring system is summarized in Table S1, Supplemental Digital Content 1, https://links.lww.com/MD/Q288.

## 3. Discussion

We report a rare case of recurrent DRESS in a 12-month-old patient triggered by PB and AMX. This case highlights the diagnostic challenges of pediatric DRESS, particularly in infants in whom such cases are rare. The patient developed fever, rash, and eosinophilia and showed positive patch test results for both PB and AMX. Although pediatric DRESS typically affects children who are approximately 9 years old,^[2]^ our case highlights a much rarer presentation in a younger patient.

AMX is widely prescribed in early childhood. For instance, a United Kingdom cohort reported that 63.9% of children receive at least 2 AMX prescription before age 2 years.^[7]^ According to the Centers for Disease Control and Prevention in the United States, AMX is the most commonly prescribed antibiotic.^[8]^ In a recent nationwide French study of 146 cases of AMX-triggered DRESS, only 1 infant, 2 children, and 4 adolescents were reported, emphasizing the rarity of DRESS in infants.^[9]^ However, none of the patients in those cases experienced a recurrence triggered by AMX re-exposure.

A previous report describing 2 adult cases documented hypersensitivity reactions to AMX following DRESS induced by carbamazepine or allopurinol; in both cases, a generalized rash and eosinophilia developed within 2 days of AMX administration.^[10]^ Although DRESS symptoms typically emerge between 2 and 8 weeks after exposure to the causative agent,^[11]^ some reports indicate that antibiotic-induced DRESS may manifest with a shorter latency period.^[12,13]^ Consequently, a symptom onset of <15 days does not exclude a DRESS diagnosis. Several studies have suggested that the onset of DRESS may be shorter with antimicrobial agents, likely due to preexisting sensitization.^[9,10,12]^ Our case findings support this possibility.

To contextualize novelty, we performed a focused PubMed search (inception to August 2025) combining DRESS, PB, and aminopenicillins. We found one pediatric report of oxcarbazepine-induced DRESS in which AMX/clavulanate was considered a possible trigger; however, neither patch testing nor DPT was performed, leaving aminopenicillin causality unconfirmed.^[14]^

We propose a progressive sensitization model to explain the recurrent DRESS episodes experienced by the patient. Initial exposure to ampicillin hydrate before the PB-induced DRESS may have primed the immune system. Subsequent re-exposure to ampicillin hydrate could then have triggered the DRESS-like symptoms seen in the second episode. The administration of AMX during that second episode may have induced either new sensitization or cross-sensitization, given its structural similarity to ampicillin. This likely cumulative sensitization contributed to the rapid onset of symptoms observed during the third episode, which occurred after administration of AMX alone.

Patch testing revealed positive reactions to PB and AMX. Since DRESS caused by ampicillin hydrate was not initially suspected, patch testing for ampicillin hydrate was not performed. In retrospect, performing a patch test for ampicillin hydrate would have improved the diagnostic accuracy in episode 2 as it would have distinguished primary sensitization from cross-sensitization. For future pediatric DRESS cases, delayed-reading skin tests for all suspected β-lactams should be considered once the patient has recovered completely. DPT should generally be avoided unless a strong clinical indication justifies the risk of relapse.

Consistent with guidelines, this case reinforces that all structurally similar β-lactams should be avoided after a DRESS episode due to the risk of progressive sensitization and cross-reactivity.^[15]^

Episode 3 showed markedly elevated TARC (33,925 pg/mL), supporting DRESS diagnosis despite a lower RegiSCAR score. Antibiotic-triggered DRESS often presents with shorter onset times, resulting in lower RegiSCAR scores. However, TARC elevation compensates for this diagnostic challenge. Normal TARC in children under 2 is 707 pg/mL^[4]^; using a 13,900 pg/mL cutoff yields 100% sensitivity and 92.3% specificity for DRESS diagnosis.^[16]^ Our patient’s value far exceeded this threshold, confirming DRESS.

Consequently, the exact role of LTT remains uncertain. In adult DRESS, LTT generally has high sensitivity and specificity,^[17]^ but its application in children is not well established.^[3]^ In this case, although LTT was negative and the patch test was positive, the DPT was positive. A negative LTT does not guarantee safety against the drug.^[17]^ Furthermore, Dhir et al recently reported 2 pediatric cases of DRESS caused by AMX/clavulanic acid. In these cases, LTT was performed on the affected children and their parents, suggesting a genetic predisposition.^[18]^ Our patient’s mother also had a history of AMX/clavulanic acid-induced rashes, suggesting a genetic predisposition. This observation highlights the potential utility of familial immune testing in such cases.

In this case, human herpesvirus 6 (HHV-6) reactivation was evaluated only at the time of onset in the third episode, as residual samples from the first and second episodes were unavailable. The evaluation of HHV-6 reactivation is highly dependent on the testing method and timing of blood collection. Although real time PCR can detect viral DNA with high sensitivity even in the early stages of onset (1–2 weeks), IgG elevation detected by antibody EIA is generally observed at approximately 2 to 3 weeks after onset, so the timing of sample collection may also affect the results.^[19]^ Moreover, the low positivity rate of HHV-6 in pediatric DRESS may help explain the negative PCR findings in this case.^[2]^ Based on these findings, we were unable to draw definitive conclusions regarding the involvement of viruses in the early stages of onset.

Large-scale investigations are essential in elucidating the characteristics and mechanisms of DRESS in infants and young children and establishing safe strategies for re-administration of implicated medications. Additional research should focus on evaluating the clinical utility of biomarkers such as TARC and refining methods for accurate detection of viral reactivation.

## 4. Conclusion

This case highlights the importance of maintaining a high index of suspicion for DRESS in infants, even when symptoms present shortly after antibiotic initiation. Given the widespread use of AMX in pediatric populations, large-scale studies are needed to better clarify the risk factors, timing, and immunological underpinnings of antibiotic-induced DRESS.

## Acknowledgments

The authors thank Yoshiki Kawamura for supporting the evaluation of HHV-6. We would like to thank Editage (www.editage.com) for English language editing and publication support.

## Author contributions

**Formal analysis:** Yurika Matsumoto.

**Funding acquisition:** Chisato Inuo.

**Investigation:** Yurika Matsumoto, Mayumi Fujita.

**Supervision:** Chisato Inuo.

**Visualization:** Yurika Matsumoto.

**Writing** – **original draft:** Yurika Matsumoto.

**Writing** – **review & editing:** Chisato Inuo.

## Supplementary Material


