# Supplementary material for: An infant with drug reaction with eosinophilia and systemic symptoms caused by phenobarbital and amoxicillin: A case report
Source: Medicine (Baltimore). 2025 Oct 10;104(41):e45034. doi: 10.1097/MD.0000000000045034 (PMC12517786; doi:10.1097/MD.0000000000045034)
Supplement: Supplementary file 1 [file medi-104-e45034-s001.pdf]

Supplemental Digital Content 1

Table S1. RegiSCAR Scoring System for the Diagnosis of DRESS (abbreviated version for quick reference)[4,5]

**Criterion**	**Assessment details**	**Score†**
Fever	≥38.5 °C (oral/core)	0
	<38.5 °C (oral/core) / U	-1
Lymphadenopathy	Enlargement in ≥2 anatomic sites	1
Eosinophilia	700–1499 cells μL□^{1} *or* 10–19% of total leukocytes	1
	≥1500 cells μL□^{1} *or* ≥20 %	2
Atypical lymphocytes	Present on peripheral□blood smear	1
Skin involvement	Rash affecting >50% body surface area	1
	Rash typical of DRESS (infiltrated erythema, facial edema, purpura, vesicles, or pustules)	1
	No Rash typical of DRESS	-1
	Skin biopsy compatible with DRESS/ U	0
	No Skin biopsy compatible with DRESS	-1
Internal□organ involvement	Single organ (e.g., ALT or AST ≥2 × ULN for ≥2 days)	1
	≥2 organs	2
Prolonged course	Signs/symptoms persisting ≥15 days	0
	Signs/symptoms persisting <15 days/ U	-1
Investigation of alternative causes	Viral, autoimmune, or neoplastic causes reasonably excluded	1

U, unknown/unclassifiable

Score interpretation

Total score (–4 to +9)	Diagnostic category
<2	Excluded
2–3	Possible
4–5	Probable
≥6	Definite
